# Overexpression of ribosomal protein L15 is associated with cell proliferation in gastric cancer

**DOI:** 10.1186/1471-2407-6-91

**Published:** 2006-04-11

**Authors:** Hui Wang, Li-Na Zhao, Kai-Zong Li, Rui Ling, Xiao-Jun Li, Ling Wang

**Affiliations:** 1 Department of Vascular and Endocrine Surgery, Xijing Hospital, the Fourth Military Medical University, 17 Changle Western Road, Xi'an 710032, China; 2 State Key Laboratory of Cancer Biology & Institute of Digestive Diseases, Xijing Hospital, the Fourth Military Medical University, 17 Changle Western Road, Xi'an 710032, China; 3 Department of Hepatobiliary Surgery, Xijing Hospital, the Fourth Military Medical University, 17 Changle Western Road, Xi'an 710032, China

## Abstract

**Background:**

Ribosomal proteins are the components of ribosome, which also exhibit various secondary functions in DNA repair, apoptosis, drug resistance and proliferation. In our previous study of microarray, ribosomal protein L15 (RPL15) was identified as an upregulated gene in gastric cancer.

**Methods:**

We investigated the expression of ribosomal protein L15 in gastric cancer and the effect of RPL15 on proliferation of gastric cancer.

**Results:**

It was found that the expression of RPL15 was markedly up-regulated in gastric cancer tissues. RPL15 was also highly expressed in gastric cancer cell lines AGS, MKN45, MKN28, SGC7901 and KATOIII. Inhibition of RPL15 expression by siRNA vector transfection suppressed the growth of SGC7901 cells significantly, which was independent of the expression of Cyclin D1 and B1. Down-regulation of RPL15 expression inhibited SGC7901 cell growth in soft agar and its tumorigenicity in nude mice.

**Conclusion:**

RPL15 promotes cell proliferation and may be a potential target for anticancer therapy of gastric cancer.

## Background

Gastric cancer is the second most common cause of cancer death worldwide. Environmental and genetic factors are both important in gastric carcinogenesis [1,2]. In recent years, knowledge about the molecular features of gastric carcinoma has increased rapidly. Loss of heterozygosity (LOH) on loci of chromosomes 1, 5, 7, 12, 13 and 17 were frequently identified in advanced gastric carcinomas [3]. Genetic alterations such as activation of oncogenes and inactivation of tumor suppressor genes could often be found in gastric cancer. It was believed that mutation or amplification of oncogenes c-ras, c-erbB-2, K-sam, c-met, and c-myc and inactivation of tumor suppressor genes p53, adenomatosis polyposis coli (APC), deleted in colorectal carcinomas (DCC), and retinoblastoma (RBI) are related to the development of gastric cancer. These genes are the potential prognostic markers to predict progression and metastasis of gastric cancer [3-5].

Recent studies have shown that comprehensive analysis of gene expression patterns can identify new molecules discriminating gastric cancer from normal gastric mucosa. This technique was also be used to distinguish tumor subgroups and identify genes whose expression may be correlated with disease classification, early detection, prognostication as well as progression of gastric cancers [6-8]. In our previous study, we compared the gene expression profiling of two gastric cancer tissues with their corresponding nontumorous tissues and found that the expression levels of ribosomal protein L15 (RPL15) in gastric cancer tissues increased 2.8- and 4.7-fold compared to those of their controls respectively (data not published). RPL15 has also been shown to be overexpressed in some esophageal tumors compared to normal matched tissues [9]. So it is interesting to see whether RPL15 is really highly expressed in gastric cancer and the effect of RPL15 on the malignant phenotype of gastric cancer. In this study, we have examined the expression in gastric cancer tissues and cell lines with the polyclonal antibody raised by ourselves. The effect of RPL15 on the growth of gastric cancer cell line SGC7901 was also investigated.

## Methods

### Tissue collection

For immunostaining of RPL15, tissue arrays was purchased from Cybrdi Co. (Chaoying Biotechnology Co., Xi'an, China), which contained 46 sections from normal gastric mucosa, gastric adenocarcinoma and adjacent nontumorous tissues respectively. For Western blotting, gastric cancer and adjacent nontumorous tissues were obtained from 12 patients who underwent surgery at the Department of General Surgery in our hospital. All cases of gastric cancer and normal gastric mucosa were clinically and pathologically proved. The protocols used in the studies were approved by the Hospital's Protection of Human Subjects Committee. Patients having fresh surgical tissue for the study signed informed consent.

### Cell lines and animals

Gastric cancer cell lines AGS, MKN45, MKN28, SGC7901, KATOIII, and SV40-transformed immortal gastric epithelial cell GES-1 were preserved in our department. Cells were routinely cultured in RPMI 1640 medium (Life Technologies, Inc., Gaithersburg, MD) supplemented with 10% fetal calf serum in a 37°C humidified incubator with a mixture of 95% air and 5% CO2. BALB/c nude mice, 4 to 6 weeks old, were provided by Shanghai Cancer Institute for the *in vivo *tumorigenicity study. The experiment was performed along established, institutional animal welfare guidelines concordant with NIH species criteria.

### Antibody

Polyclonal antibodies were prepared against a peptide (C) RVRCWQYRQLSALHR from the sequence of RPL15. Antibodies were commercially prepared by immunization of 2 rabbits (Biosynthesis Biotechnology Co., Beijing, China). The antibody was affinity purified against the peptide on a Sulfolink column (Pierce) and used at 2 μg/ml and 6 μg/ml in western blot and immunohistochemistry respectively.

### Immunohistochemistry

Tissue arrays were dewaxed, rehydrated, incubated in 10% normal goat serum and 0.3% Triton X-100 in PBS for 1 hr and then incubated with anti-RPL15 antibody. The arrays were washed in PBS 3 times for 5 min each. The tissues were incubated in biotin-labeled goat anti-mouse serum (1:200) for 30 min, rinsed with PBS and incubated with avidin-biotin-peroxidase complex for 1 hr. The signal was detected using 3,3-diaminobenzidine as the chromogen. Only cells with brown-colored cytoplasmic staining were considered as positive. Result was evaluated by formula as described previously [10]: the staining score = the intensity of immunoreactivity (IR) × the proportion of positively staining cells. The intensity of IR was stratified into 4 categories: 0, no IR; 1, weak IR; 2, moderate IR and 3, strong IR. The proportion of positive cells was classified into 4 groups: 0, 0–5% tumor cells exhibiting IR; 0.33, 5–33% of the tumor cells exhibiting IR; 0.67, 33–67% of the tumor cells exhibiting IR and 1, 67–100% of the tumor cells exhibiting IR.

### Plasmid construction

For the construction of pSilencer-RPL15A and pSilencer-RPL15B, two pairs of oligonucleotides were respectively synthesized as follows: P1: 5'-GAT CCA GAA GCA GTC TGA TGT CAT TAC AAG AGA ATG ACA TCA GAC TGC TTC TTT TTT TGG AAA-3'; P1': 5'-AGC TTT TCC AAA AAA AGA AGC AGT CTG ATG TCA TTC TCT TGT AAT GAC ATC AGA CTG CTT CTG-3'; P2: 5'-GAT CCG GTT ACG TTA TAT ATA GGA TTC AAG AGA TCC TAT ATA TAA CGT AAC CTT TTT TGG AAA-3'; P2': 5'-AGC TTT TCC AAA AAA GGT TAC GTT ATA TAT AGG ATC TCT TGA ATC CTA TAT ATA ACG TAA CCG-3'. The two oligonucleotides were annealed and cloned into pSilencer3.0 (Ambion, Austin, TX), respectively. The insert sequences were confirmed by DNA sequencing.

### Cell transfection

Cell transfection were performed with Lipofectamine 2000 (Invitrogen, Carlsbad, CA) as described in the manufacturer. Briefly, SGC7901 cells were plated and grown to 80–90% confluence without antibiotics. Then they were transfected with 1 μg siRNA expressing vector pSilencer-RPL15A or pSilencer-RPL15B. After 24 h of transfection, G418 (400 μg/ml) was added into cells for stable screening. The mixed clones were expanded for an additional 2 months. Cells transfected with pSilencer-RPL15A, pSilencer-RPL15B or empty vector pSilencer3.0 were designated as SGC7901-L15AsiRNA, SGC7901-L15BsiRNA and SGC7901-pcDNA respectively.

### Western blot

Cells grown to 70–90% confluence were collected by scraping and washed with ice-cold PBS for two times. The cell pellets or tissues were homogenized in extraction buffer (50 mM Tris-HCl, 0.1%SDS, 150 mM NaCl, 100 μg/ml phenylmethylsulfonyl fluoride, 1 μg/ml aprotinin, 1% NP-40, and 0.5% sodium orthovanadate), then incubated at 4°C for 30 min, centrifuged 20 min at 12,000 g/min. Concentration of total proteins in the supernatant was quantified by Bradford assay. The total proteins (50–100 μg/lane) were resolved in 8–12% SDS-polycrylamide gels, then transferred onto nitrocellulose membrane (0.45 μm, Millipore, USA) in 25 mM Tris-base, 190 mM glycine, and 20% methanol using a semi-dry blotter. Following blocking with 8% fat-free milk and 0.1% Tween20 in TBS for 2 h, the membrane was incubated with anti-RPL15, anti-Cyclin D1(Neomarker, 1:200), anti-Cyclin B1(Neomarker, 1:200) and anti-β-actin(Sigma, 1:5000) at 4°C overnight. After binding of horseradish peroxidase (HRP)-coupled goat anti-mouse or goat anti-rabbit IgG (Santa Cruz, 1:5000) at room temperature for 2 hours, antigens were visualized by enhanced chemiluminescence (ECL-kit, Santa Cruz Biotechnology). All results are representatives of three independent experiments.

### MTT assay

3-(4,5-dimethylthiazol-2-yl)-2,5-diphenyl-tetrazolium bromide assay was performed to evaluate the speed of cell proliferation as described before [11]. Briefly, log phase cells were trypsinized into single cell suspension and passaged into 96-well plates at a density of 1 × 10^3 ^cells/well. The total volume of culture medium in each well was 200 μl. After 1 d, 2 d, 3 d, 4 d and 5 d of cell culture, MTT solution was added into each well. After 4 hrs, the supernatant was carefully removed. 150 μl DMSO was used to dissolve the crystals by agitation for 10 min. The absorbance at 490 nm (A490) of each well was read on a microplate reader BP800 (BIOHIT). Each study was performed in quadruplicate and repeated 3 times.

### Flow cytometry

Cells in log phase were washed twice with PBS and fixed with 70% ethanol, washed again, and resuspended at 37°C for 15 min in PI staining solution (PBS containing 0.1% (v/v) Triton X-100 (Sigma), 0.2 mg/ml Dnase-free RNase A (Roche), and 20 μg/ml PI), prepared fresh daily. 1 × 10^4 ^cells were then analyzed for fluorescence intensity by FACScan (BD Biosciences) using the CellQuest flow-cytometric analysis software. Cell proliferation index is calculated with the formula: PI (proliferation index) = number of S, G2 and M stage cells/number of total cells ×100%. Each study was repeated 4 times.

### Soft agar clonogenic assay

Anchorage-independent growth as a characteristic of in vitro tumorigenicity was assessed by soft agar clonogenic assay described previously [11]. Briefly, cells were detached and plated in 0.3% agarose with a 0.5% agarose underlay (1 × 10^3^/well in 6-well plates). The number of foci >100 μm in 10 randomly selected field was counted under microscope after 20 days. Each study was performed in triplicate and repeated 3 times.

### Mouse experiments

Mice were handled using best humane practices and were cared for in accordance with NIH Animal Care and Use Committee guidelines. Cells were harvested from tissue culture flasks using trypsin and resuspended in PBS. Mice were injected subcutaneously with 2 × 10^6 ^cells in 0.1 ml into the right upper back and raised in the following 30 days. The mice were then monitored for tumor volume, overall health, and total body weight. The size of the tumor was determined by caliper measurement of the subcutaneous tumor mass. Tumor volume was calculated according to the formula 0.5 × length × width^2^. Each experimental group contained 6 mice. Two independent experiments were performed and gave similar results.

### Statistical analysis

Bands from Western blot or RT-PCR were quantified by Quantity One software (BioRad). Relative protein or mRNA levels were calculated by referring them to the amount of β-actin. Numerical data are presented as the mean ± SEM. The difference between means was performed with Student's T test. Immunohistochemical results were analyzed by Kruskal-Wallis rank test and the Mann-Whitney U test. All statistical analyses were performed using SPSS11.0 software (Chicago, IL, USA). p < 0.05 was considered as statistically significant.

## Results

### Expression of RPL15 in gastric cancer tissues and cell lines

**Figure 1:**
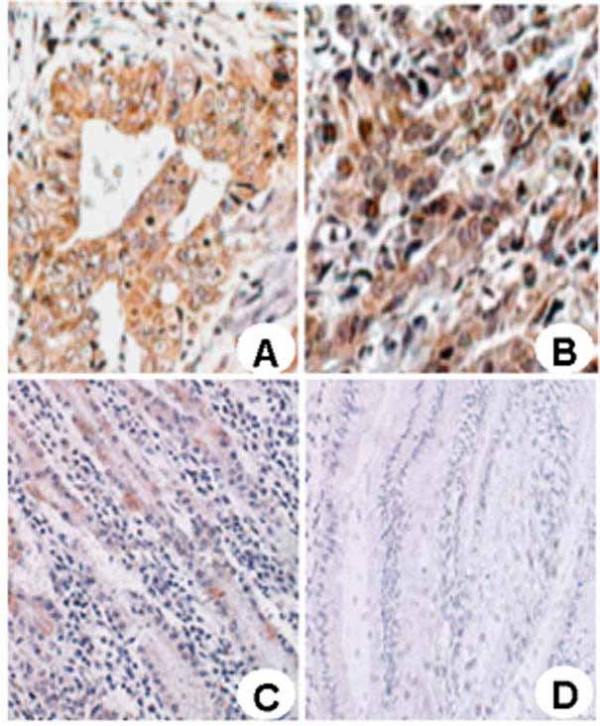
**Expression of RPL15 in normal gastric mucosa, gastric cancer and adjacent nontumorous tissues**. Immunohistochemical staining was preformed to evaluate RPL15 expression in gastric carcinoma and normal gastric mucosa tissues (×200). (A) Well-differentiated gastric carcinoma; (B) Poorly-differentiated gastric carcinoma; (C) adjacent nontumorous tissue; (D) Normal gastric mucosa.

To examine whether the RPL15 expression level is altered in gastric cancer, the expression and subcellular localization of RPL15 were studied in a set of gastric cancer patient specimens derived from 23 normal gastric mucosae (NG), 23 gastric cancer (GC) and 23 adjacent nontumorous tissues (NT). RPL15 was found expressed predominantly in the cytoplasm of cells in gastric mucosa and cancer (Fig. 1). The RPL15 staining in epithelial cells from both NG and NT samples were weak, and the average staining scores of NT was 2.8 ± 1.13, higher than 1.2 ± 0.49 in NG (*p *< 0.05). The RPL15 staining in gastric cancer cells was consistently stronger than those of the NG and NT samples (Figs. 1A and 1B) with an average score 6.3 ± 2.90 (*p *< 0.01). To investigate if the RPL15 expression might be associated with the progression of gastric cancer, the relationship between the RPL15 expression levels and the clinicopathologic characteristics of different gastric cancer patients was compared. The result showed that RPL15 expression did not correlate with differentiation grade, Tumor-Node-Metastasis stage, gross type, or metastasis with a statistic *p *> 0.05 in each parameter (data not shown). These results provide evidence that RPL15 expression level positively correlates with gastric cancer onset but is not associated with the progressiveness of the cancer.

### Analysis of RPL15 expression in gastric cancer cells and tissues

**Figure 2:**
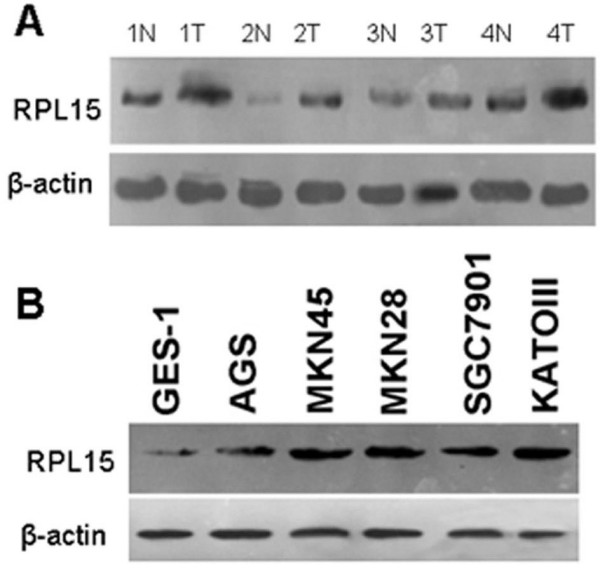
**Western blot analysis of RPL15 expression in gastric cancer tissues and cell lines**. A, protein isolated from gastric cancer and adjacent nontumorous tissues obtained from 12 patients was separated by SDS-PAGE and subjected to western blot analysis using anti-RPL15 and anti-β-actin antibodies. Four representative pairs of tissues are presented. N, normal tissue; T, gastric cancer tissue. B, GES-1 and five gastric cancer cells were collected and lysed, then analyzed by Western blotting. The level of RPL15 expression was much higher in all five gastric cancer cell line than in GES-1.

Expression levels of RPL15 were examined by western blot in the gastric cancer and adjacent normal tissues taken from 12 patients (Fig. 2A). In 9 of the 12 cases or 75% samples, RPL15 was found overexpressed in cancerous tissues, consistent with the result from immunohistochemistry analysis. We further compared the relative RPL15 expression level in five different gastric cancer cell lines, i.e., AGS, MKN45, MKN28, SGC7901, and KATOIII, with that of the normal gastric cell line GES-1. Again, RPL15 was found expressed at a higher level in all five gastric cancer cell lines than in GES-1 cells (Fig. 2B). These results suggest that RPL15 may have a role in gastric carcinogenesis.

### Interference of RPL15 expression inhibited SGC7901 cell proliferation

**Figure 3:**
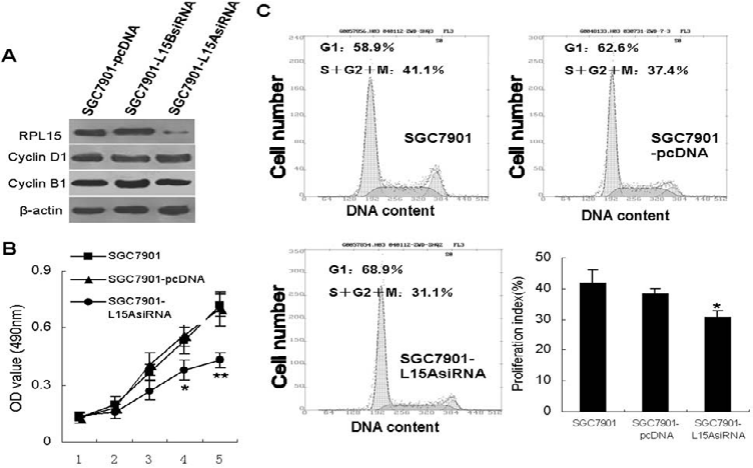
**Effect of RPL15 expression on cell proliferation**. A, SGC7901 cells transfected with pSilencer-RPL15A, pSilencer-RPL15B or empty vector were selected with G418 (400 ng/ml) for 2 months. The mixed clones were used for analyzing the expression of RPL15, Cyclin B1 and Cyclin D1 by western blot. Anti-β-actin antibody was set as an internal control. B, Cell proliferation was determined by MTT assay. Cells stably transfected with pSilencer-RPL15A or empty vector were plated in quadruplicate in 96-well plates. At the indicated time intervals, the monolayer cells were processed for MTT assay and the absorbance was read at 490 nm in an enzyme-linked immunosorbent assay reader. **p < 0.05 and *p < 0.01 *vs *SGC7901 and SGC7901-pcDNA. C, cell cycle distribution of log-phase transfected cells was evaluated by FACScan. Cell proliferation index was calculated as described in Materials and Methods (low right panel). Each number represents mean ± SD of four different experiments. *p < 0.01 *vs *SGC7901 and SGC7901-pcDNA.

To further analyze the involvement of RPL15 in gastric cancer malignant phenotype, we took advantage of siRNA technology. SGC7901 cells were transfected with pSilencer-RPL15A, pSilencer-RPL15B or empty vector. After G418 selection for 2 months, western blot analysis revealed that the cell mixture transfected with pSilencer-RPL15A instead of pSilencer-RPL15B showed substantial reduction in RPL15 level (Fig. 3A).

We further investigate the effect of pSilencer-RPL15A transfection on cell growth of gastric cancer. The result of MTT showed that SGC7901-L15AsiRNA cells grew significantly slower than the control cells (SGC7901 and SGC7901-pcDNA; Fig. 3B). Cell cycle analysis revealed that the population of cells in the S+G2+M phase was remarkably decreased by pSilencer-RPL15A expression (31.1% of asynchronous SGC7901-L15AsiRNA cells in the S+G2+M phase of the cell cycle compared with 41.1% of control cells and 37.4% of SGC7901 cells, Fig. 3C). We questioned if RPL15 promote cell cycle progress by changing the expression of cyclins. Western blot demonstrated that neither expression of Cyclin D1 nor Cyclin B1 were influenced by ectopic expression of RPL15 (Fig. 3A), indicating there might be other molecules mediating the promoting effect of RPL15 on cell proliferation of gastric cancer.

### Interference of RPL15 expression or activity inhibited tumorigenicity of SGC7901 Cells

**Figure 4:**
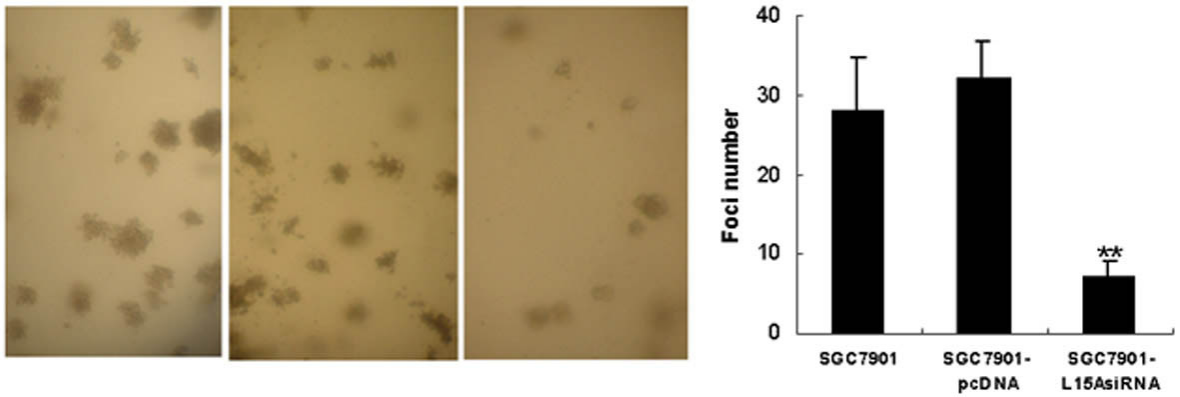
**Interference of RPL15 expression inhibits colony formation ofSGC7901 cells**. Cells were placed in media containing soft agar and incubated for 20 days. The number of foci >100 μm was counted in 10 random fields. Vertical bars (right panel) represent mean ± SD from three separate experiments, each conducted in triplicate. **p < 0.05 *vs *SGC7901 and SGC7901-pcDNA.

**Figure 5:**
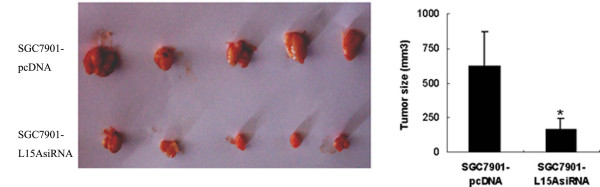
**Tumorigenicity ability of SGC7901 cells transfected with siRNA vectors. Tumorigenicity ability was evaluated by nude mice assay**. Mice were injected subcutaneously with 2 × 10^6 ^transfected cells. After 1 month of cell injection, the volume of tumor was calculated (left panel) according to the formula 0.5 × length × width^2^. Two independent experiments were performed. *p < 0.05 vs SGC7901-pcDNA.

Anchorage-independent growth could be a sensitive marker for tumor growth in vivo; therefore, we examined whether interference of RPL15 expression would cause an inhibition of SGC7901 cell growth in soft agar. As shown in Fig. 4, siRNA transfection resulted in a substantial reduction in the colony number (5–9 colonies, respectively) when compared with the control cells (SGC7901-pcDNA and SGC7901, 25–35 and 28–39, both p < 0.01). To confirm this effect in vivo, we injected subcutaneously the siRNA expressing SGC7901 cells into the right upper back of athymic nude mice to see if they might affect tumor induction. Fig. 5 shows the relative tumor growth for two cell lines. On experiment day 30, the average tumor volume in control animals was 628.3 mm^3 ^compared to 157.4 mm^3 ^in SGC7901-L15AsiRNA group.

## Discussion

A ribosome is an organelle composed of rRNA and two subunits, which can work together as a factory to translate mRNA into a polypeptide chain during protein synthesis in cell. In numerous cancers, it has been found that protein synthesis rates and the expression of several translation components are often significantly elevated, indicating the potential importance of ribosome function and translational control in tumor progression[12,13].

At present, approximately over 80 ribosomal proteins are found to localize within the large and small subunits of eukaryotic ribosomes[14]. Some of these ribosomal proteins are differentially or ectopically expressed in cancer cells as compared to normal cells. Kasai et al determined the expression profile of 12 ribosomal proteins in colorectal cancer and normal epithelia of human colorectal mucosa using immunohistochemistry [15]. They found that the expression of 10 ribosomal proteins (Sa, S8, S12, S18, S24, L13a, L18, L28, L32 and L35a) were decreased and 2 proteins (S11 and L7) were significantly enhanced in colorectal cancers. Results of real time RT-PCR showed that RPL44 mRNA was overexpressed in hepatocellular carcinoma tissues and cell lines [16]. Additionally, expression of S8, L12, L23a, L27 and L30 ribosomal protein mRNAs was up-regulated in human hepatocellular carcinoma [17]. Another ribosomal protein gene ectopically expressed in cancer is RPL14, transcriptional loss of which was observed in lung and oral cancers [18]. All these data indicated that the ectopic expression of ribosomal proteins be a common phenomenon in the development of cancer. In the present study, for the first time we reported that RPL15 was highly expressed in gastric cancer tissues and cell lines, indicating that RPL15 serve as a potential marker for diagnosis of gastric cancer. It is becoming increasingly clear that a growing number of ribosomal proteins exhibit various secondary functions besides protein synthesis[12,13]. There have been many reports that implicate ribosomal proteins in developmental or regulatory processes. There is evidence that ribosomal proteins may be tumor suppressors, oncogenes and gene-specific translational regulators. Roles for ribosomal proteins in DNA repair and regulation of transcription have been proposed [9,19] Ribosomal proteins are also involved in regulation of apoptosis. It has been reported that ribosomal protein S29 induces apoptosis in non-small cell lung cancer cells by downregulation of Bcl-2, Bcl-XL and survivin and upregulation of p53, and Bax [20]. And human ribosomal protein L13a also induces apoptosis, presumably by arresting cell growth in the G2/M phase of the cell cycle [21].

In the present study, we demonstrated that inhibition of RPL15 expression could suppress the proliferation of gastric cancer by in vitro and in vivo assay. These results suggested that RPL15 promote the growth of gastric cancer, similar to the results of Kim et al in liver cancer. They reported an HCC-related gene, RPL44, which can enhanced colony formation and cell proliferation of HCC cells [16] However, no molecular mechanisms in detail had been explored in both of these two studies. So further work to elucidate why some ribosomal proteins can promote the proliferation of tumor cells should be done in the future.

## Conclusion

RPL15 is highly expressed in gastric cancer tissues and cell lines. It plays a role in cell proliferation of gastric cancer. So RPL15 may be a potential target for anticancer therapy of gastric cancer.

## Competing interests

The author(s) declare that they have no competing interests.

## Authors' contributions

Ling WANG analyzed the results, and prepared the manuscript for submission;

Hui WANG was involved in design of the studies and perform plasmid construction and cell transfection;

Li-Na ZHAO was involved in cell culture, animal experiment and discussion of the results.

Kai-Zong LI was involved in design of animal studies, analyses of the data and their interpretation as well as writing the manuscript.

Rui LING was involved in perform flow cytometry.

Xiao-Jun LI was involved in perform MTT assay.

## Pre-publication history

The pre-publication history for this paper can be accessed here:
